# Exploring the Combined Action of Adding Pertuzumab to Branded Trastuzumab versus Trastuzumab Biosimilars for Treating HER2+ Breast Cancer

**DOI:** 10.3390/ijms25073940

**Published:** 2024-04-01

**Authors:** Emma Franco-Mateos, Virginia Souza-Egipsy, Laura García-Estévez, José Pérez-García, María Gion, Laia Garrigós, Patricia Cortez, Cristina Saavedra, Patricia Gómez, Carolina Ortiz, Víctor L. Cruz, Javier Ramos, Javier Cortés, Juan F. Vega

**Affiliations:** 1 BIOPHYM, Department of Macromolecular Physics, Instituto de Estructura de la Materia, IEM-CSIC, C/Serrano 113 bis, 28006 Madrid, Spain; emmafranco00@gamil.com (E.F.-M.); virginia.souza-egipsy@csic.es (V.S.-E.); vl.cruz@csic.es (V.L.C.); j.ramos@csic.es (J.R.); 2 Breast Cancer Department, MD Anderson Cancer Center, 28033 Madrid, Spain; lgestevez@mdanderson.es; 3 International Breast Cancer Center (IBCC), Pangaea Oncology, Quiron Hospital, 08017 Barcelona, Spain; josemanuel.perez@ibcc.clinic (J.P.-G.); laia.garrigos@ibcc.clinic (L.G.); patricia.gomez@ibcc.clinic (P.G.); carolina.ortiz@ibcc.clinic (C.O.); javier.cortes@maj3.health (J.C.); 4 Medica Scientia Innovation Research (MedSIR), 08018 Barcelona, Spain; 5 Medica Scientia Innovation Research (MedSIR), Ridgewood, NJ 07450, USA; 6 Medical Oncology Department, Ramón y Cajal University Hospital, 28034 Madrid, Spain; mariagion@gmail.com (M.G.); cristina.saavedra@iobmadrid.com (C.S.); 7 IOB, Institute of Oncology, 28007 Madrid, Spain; patricia.cortez@iobmadrid.com; 8 Faculty of Biomedical and Health Sciences, Department of Medicine, Universidad Europea de Madrid, 28670 Madrid, Spain

**Keywords:** HER2^+^ breast cancer, monoclonal antibodies, trastuzumab biosimilars

## Abstract

The binding activity of various trastuzumab biosimilars versus the branded trastuzumab towards the glycosylated extracellular domain of the human epidermal growth factor receptor 2 (HER2) target in the presence of pertuzumab was investigated. We employed size exclusion chromatography with tetra-detection methodology to simultaneously determine absolute molecular weight, concentration, molecular size, and intrinsic viscosity. All trastuzumab molecules in solution exhibit analogous behavior in their binary action towards HER2 regardless of the order of addition of trastuzumab/pertuzumab. This analogous behavior of all trastuzumab molecules, including biosimilars, highlights the robustness and consistency of their binding activity towards HER2. Furthermore, the addition of HER2 to a mixture of trastuzumab and pertuzumab leads to increased formation of high-order HER2 complexes, up to concentrations of one order of magnitude higher than in the case of sequential addition. The observed increase suggests a potential synergistic effect between these antibodies, which could enhance their therapeutic efficacy in HER2-positive cancers. These findings underscore the importance of understanding the complex interplay between therapeutic antibodies and their target antigens, providing valuable insights for the development of more effective treatment strategies.

## 1. Introduction

Monoclonal antibodies (mAbs) have revolutionized cancer treatment over recent years, offering precise targeting to specific proteins or cells. In the case of breast cancer, several mAbs-based therapies have been developed and approved for use [1]. Notable among these are trastuzumab and pertuzumab, and more recently tucatinib and trastuzumab deruxtecan [2]. These drugs target the very aggressive human epidermal growth factor receptor 2-positive (HER2+) cancer cells by blocking the action of the HER2 protein [3,4]. The HER2 transmembrane protein is a member of the family of epithelial growth factor receptors, the overexpression of which is related with aggressive carcinomas. Presently, the 5-year survival rate is increasing up to 90% in HER2+ early breast cancer treated with chemotherapy and combined or dual mAbs trastuzumab and pertuzumab therapies, as they show complementary mechanisms of action [5].

Clinical trials and meta-analyses have reported cases of sequential addition in patients undergoing dual anti-HER2 therapies. Jagosky and Liu [6,7] have recently reviewed several studies that support the substantial improvement in the outcome of HER2+ breast cancer patients with combined therapy of pertuzumab and trastuzumab [8,9,10,11,12,13,14,15,16,17]. 

Additionally, it has been demonstrated that the combination of trastuzumab emtansine (T-DM1) and pertuzumab exhibits synergistic cytotoxic activity in cell culture and enhanced antitumor activity [18]. Moreover, the results indicated that the safety of dual-target therapy is similar to that of single-target therapy [11,19]. 

On the other hand, biosimilars highly resemble the branded drugs, but with the advantage of quicker development, lower cost of production, and greater availability. As the development process of a biosimilar is somewhat different to that of the reference product, it is possible to include minor changes or additions to the protein structure that can produce a drug with different efficacy or side effect profiles. However, a biosimilar drug may not be an exact copy of the reference product, but it must maintain identical or even improve its mechanisms of action, dosage and route of administration, and effectiveness [20,21,22]. Several biosimilars of Herceptin^®^ [Genentech, Inc., South San Francisco, CA, USA] have been approved for use in breast cancer treatment, including Ontruzant^®^ [N.V. Organon, Jersey City, NJ, USA] and Herzuma^®^ [Celltrion, Inc., Inchon, South Korea] [23,24,25]. 

In this study, we assessed the ability of each of these antibodies to bind to HER2 in the presence of the pertuzumab Perjeta^®^ [Genentech, Inc.], and investigated the order of addition of the mAbs to HER2 in solution. The selected methodology for investigating the binding process was size exclusion chromatography with tetra-detection (SEC-TD) in solution. We acknowledge that alternative techniques, such as nuclear magnetic resonance, isothermal titration calorimetry, and analytical ultracentrifugation, could potentially yield complementary results. This analytical approach offers numerous advantages for examining protein–protein interactions or the binding between mAbs and target proteins. SEC-TD is fast, efficient, cost-effective, and sensitive, enabling the simultaneous detection of proteins and complexes in a single analytical run. Additionally, it has been reported that binding experiments in solution may yield more realistic results compared to protocols using antibody support techniques [26]. This approach facilitates the measurement of composition, absolute molecular weight and size, concentration, and molecular density or intrinsic viscosity of biomacromolecules, providing a comprehensive overview of protein-protein interactions and the stability of formed complexes [27,28,29,30,31]. To the best of our knowledge, such vital information is lacking in the scientific literature, thereby constituting an essential test for assessing the biophysical comparison of biosimilar antibodies. Our findings could provide insights into potential combination therapies for HER2+ breast cancer and contribute to the optimization of treatment efficacy.

## 2. Results

The hydrodynamic and electrostatic characterization results of the mAbs are presented in Figures S1–S3 and Tables S1 and S2 of the Supplementary Material archive. The findings depicted in Figures S1–S3 indicate a considerable level of resemblance among the examined mAbs. Specifically, the diffusion coefficient (representing hydrodynamic size) exhibits variances of no more than 2% across the samples, indicating a closely matched, if not indistinguishable, morphology or shape of the mAbs. The reported positive value of the net charge, Z, for the mAbs at pH 7.5 is in the same range as those values reported in other studies for IgG1 antibody at pH within 5.0 and 9.0 and low ionic strength. A difference is found between trastuzumab and pertuzumab, explained by the differences in their amino acid sequences (AAS). No appreciable differences have been found between trastuzumab and the two biosimilars, Herzuma and Ontruzant, in this aspect. The thermal stability results, as depicted in Figures S4 and S5, yielded valuable insights into the capacity of the mAbs to uphold their structural integrity and functionality across a spectrum of temperatures. Notably, no discernible disparities in stability were observed among the various antibodies tested, indicating consistent resilience across the antibody samples.

**Figure 1:**
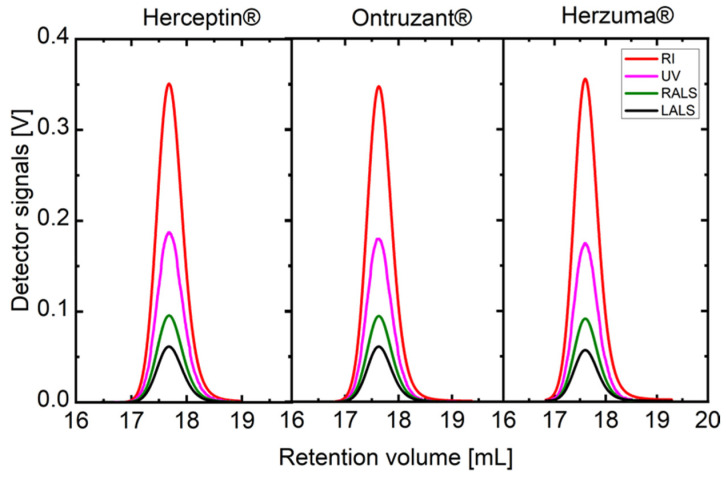
SEC traces for the trastuzumab Herceptin and the biosimilars Ontruzant and Herzuma. Four detectors—refractive index: RI; ultraviolet: UV; right-angle light scattering: RALS; and low-angle light scattering: LALS—of the biosimilars studied for a concentration of 1.0 mg·mL^−1^ at a temperature of T = 309 K are indicated.

Herceptin and the biosimilars of trastuzumab, namely, Ontruzant and Herzuma, exhibit identical SEC-TD profiles, as demonstrated in Figure 1. Table 1 outlines the main molecular and physical–chemical characteristics of the mAbs and the glycosylated extracellular domain of HER2 (g-eHER2) utilized in this study. These properties were acquired from the SEC-TD outcomes depicted in Figure 1 and dynamic light scattering (DLS) experiments, conducted at a temperature of T = 309 K (36 °C) as described in the Methods section (see also Supplementary Material, in which the complete characterization of the mAbs is described). 

In this study, we investigated the binding activity of trastuzumab biosimilars with the HER2 receptor in both binary and ternary interactions. Firstly, we examined the interactions involving either HER2 with trastuzumab or HER2 with pertuzumab individually. Subsequently, we explored the synergistic effects of combining both trastuzumab and pertuzumab antibodies with the HER2 receptor. To analyze these interactions, we introduced HER2 to an excess of monoclonal antibodies (mAbs) in three different scenarios: (1) adding HER2 to trastuzumab followed by pertuzumab, (2) adding HER2 to pertuzumab followed by trastuzumab, and (3) adding HER2 to a 1:1 mixture of both mAbs. Cases (1) and (2) involved a two-step process, first the binary interaction followed by the ternary interaction, while case (3) focused solely on the ternary interaction. In all cases, we maintained a consistent mAbs/HER2 ratio of 3:1. Molecular features of the resulting complexes from each step, including binary (C1 and C2 complexes) and ternary (C3 complex) interactions, were measured and presented in Table 2, Table 3 and Table 4.

**Figure 2:**
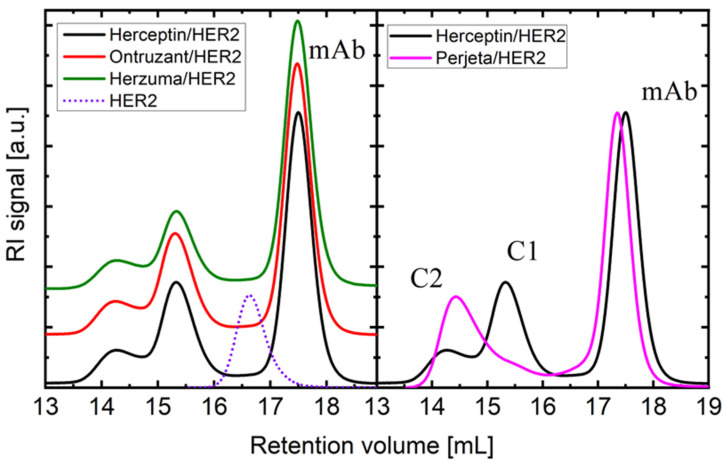
SEC traces of mAb/HER2 complexes in binary interaction. SEC traces (RI signal) of complexes between each trastuzumab biosimilar and HER2 (**left**), and comparison of trastuzumab/HER2 and pertuzumab/HER2 traces obtained in the same conditions (**right**).

**Figure 3:**
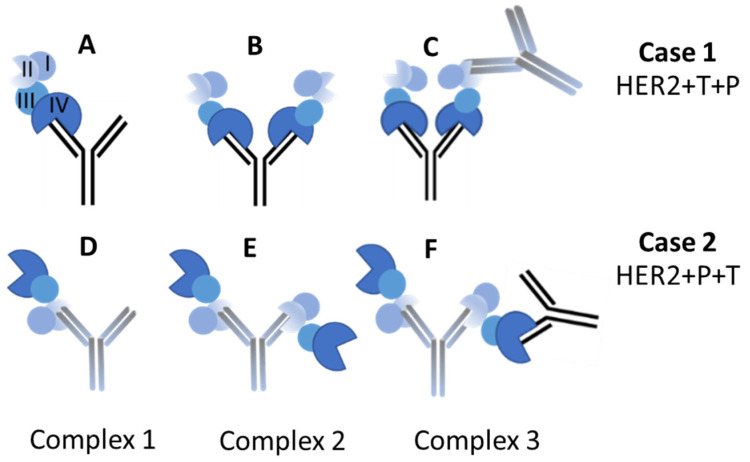
Schematic representation of the different complexes: HER2 extracellular domains (blue), trastuzumab (T, black) and pertuzumab (P, grey). The different HER2 domains are indicated in (**A**). (**A**,**D**) represents the complex 1 formed by trastuzumab and pertuzumab with HER2, respectively; (**B**,**E**) represent the complex 2 for tratuzumab and pertuzumab with HER2, respectively; and (**C**,**F**) represent the Complex 3, in which both mAbs are involved.

It is important to note that the binding affinity of trastuzumab and pertuzumab in their binary interaction to HER2 differs in solution (Figure 2). When HER2 is added to an excess of trastuzumab, it mainly forms a heterodimer (C1, one HER2 domain bound to one mAb Fab, Figure 3A) with a molecular weight of M_w_ = 235.0–238.0 kDa. Additionally, a HER2/mAb/HER2 heterotrimer (C2, two HER2 bounds to the two available mAb Fabs, Figure 3B) is formed with M_w_ = 310–316 kDa. On the other hand, pertuzumab is more likely to form the heterotrimer (C2, Figure 3E) with both Fabs of the mAb bound to two HER2 copies, resulting in a value of M_w_ = 310.0 ± 5.0 kDa (Figure 2, right panel). The biosimilars Ontruzant and Herzuma showed identical profiles to Herceptin in the binding binary step with HER2 extracellular domain, as they also formed mostly the same complexes in nearly identical proportions, as shown in Figure 2 (left panel). We measured the sizes of the two complexes (C1 and C2) to be different, around 6.2 and 7.3 nm, respectively (see Table 2 and Table 3).

We acknowledge that there are slight differences in the reported values of M_w_ for different samples of complexes C1 and C2 in Table 2. While the range for each complex is indeed relatively narrow (between 235.7 and 238.2 kDa for C1 and between 310.8 and 315.7 kDa for C2), there are variations that fall within the margin of accepted experimental variability (less than 2%). This small variability in M_w_ does not significantly impact the hydrodynamic radius values reported in our study. This observation is particularly pertinent due to the compact conformation of the proteins, which minimizes changes in hydrodynamic properties despite minor fluctuations in molecular weight. Furthermore, we note that the hydrodynamic radius values for both C1 and C2 complexes are consistent with the values of the molecular weight, with reported sizes of 6.2 nm and 7.2 nm, respectively.

**Figure 4:**
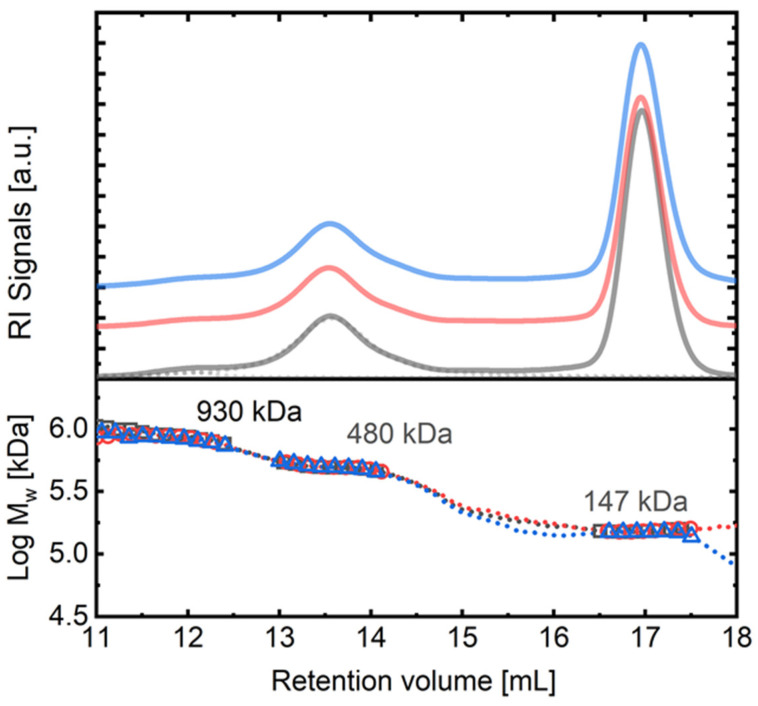
Comparison of the SEC traces obtained for the complexes in case 2 for ternary interaction. Upper panel: complete SEC-IR traces of the complexes and free mAb for case 2: black (HRC/HER2/PJT), red (ONT/HER2/PJT), and blue (HZM/HER2/PJT). Curves have been vertically shifted for better visualization. Bottom panel: measured absolute molecular weight obtained for the complexes.

In the next step of our study, by adding a second antibody to the binary solution for the ternary interaction, larger complexes in all three cases are obtained (around a retention volume of 13.5 mL). The M_w_ of these complexes is around 480–490 kDa, and there are no differences found in the case of the biosimilars at this point. Figure 4 exemplifies this result, showing case 2 of addition using pertuzumab (Perjeta) plus trastuzumab, Herceptin, and the biosimilars. The main complex found at 13.5 mL has a molecular weight that is consistent with the association of two antibodies (trastuzumab and pertuzumab) linked through an HER2 copy, with the other Fab of pertuzumab also linked to a HER2 protein unit (C3, see Figure 3F). Note that pertuzumab and trastuzumab bind to different HER2 domains (domain II and IV, respectively).

The production of the C3 complex with a molecular weight of M_w_~480–490 kDa was examined in case 1, case 2, and case 3 experiments. The concentration of the C3 heterocomplex over the total concentration of HER2 added was determined based on RI detector signals, and it was found that the differences in the production of the C3 complex for case 2 experiments (Figure 4) with the different trastuzumab biosimilars were smaller than 1%. The same trend was observed in case 1. Interestingly, in both cases 1 and 2, a very small percentage (around 1–3%) of a very high molecular weight complex (M_w_~930 kDa) was also detected around a retention volume of 12 mL.

**Figure 5:**
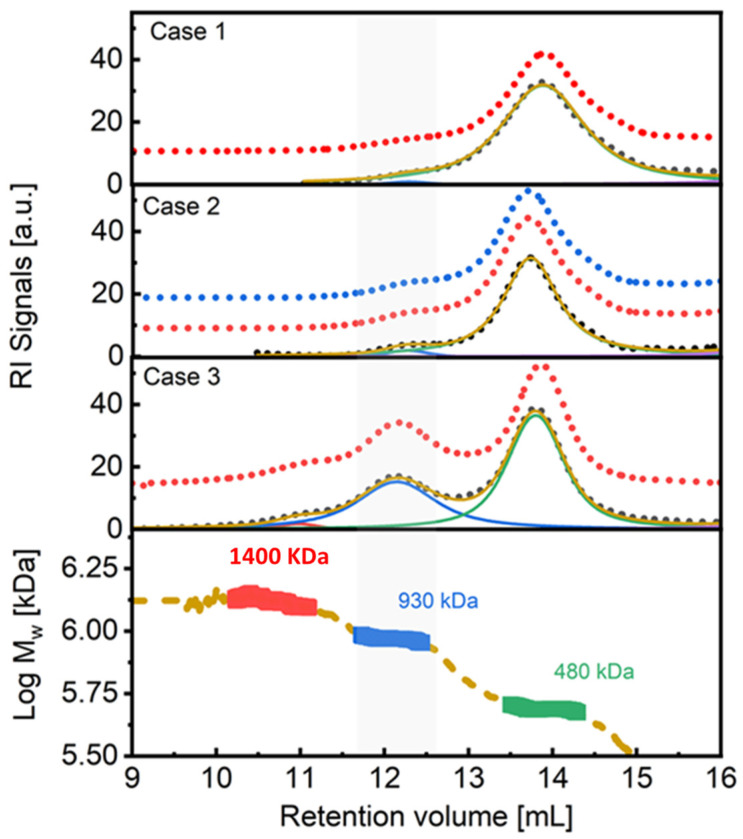
Comparison of the SEC traces obtained for the complexes in case 1, 2, and 3 for ternary interaction. Upper panels: SEC-IR traces of the complexes formed in the different cases under study. Bottom panel: Measured absolute molecular weight obtained for the complexes. C3 complex appears around 12 mL (grey zone).

In case 3 experiments, for which HER2 was added to a 1:1 mixture of trastuzumab and pertuzumab in a direct ternary interaction, a significant increase in the concentration of high-order complexes of M_w_ = 930–940 kDa was measured, appearing at a retention volume of 12 mL. The increase in the production of these complexes, with respect to cases 1 and 2, was over one order of magnitude (30%). Figure 5 provides a visual comparison of the results obtained in cases 1, 2, and 3. The signals have been deconvoluted in order to extract the concentration of each species. It is evident that a higher amount of high molecular weight complexes was formed in case 3, and even a small amount of very high molecular weight complexes of around 1400 kDa was detected, around a retention volume of 10–11 mL.

## 3. Discussion

Previous works by our group presented a complete experimental and computational modelling study of the molecular and hydrodynamic details of both HER2 and mAbs, which agree with the results summarized in Table 1 [32,33]. Based on the results presented, all proteins in this study exhibit the expected molecular and hydrodynamic properties consistent with their AAS. In addition to the basic molecular and hydrodynamic characterization, we have determined the extinction coefficient, dA/dc, of the proteins studied using the UV signal to match protein concentration with that measured from the RI detector. Trastuzumab biosimilars (1.38 ± 0.02 mL·g^−1^) display a slightly higher value of dA/dc compared to pertuzumab (1.33 ± 0.02 mL·g^−1^), as expected due to the higher number of aromatic residues in the former. Although both trastuzumab and pertuzumab show a high degree of chemical similarity, there are subtle differences in the amounts of tyrosine (Tyr), tryptophan (Trp), and phenylalanine (Phe) residues, which can affect the extinction, ε, or absorption coefficient, dA/dc, as measured by UV spectroscopy. The extinction coefficient at 280 nm is unique to each protein and depends on the number of aromatic residues as well as the solvent, temperature, and pH. We used the Protein Calculator Resource (http://protcalc.sourceforge.net/, accessed on 31 March 2024) to theoretically compute dA/dc for the samples under study, utilizing the corresponding AAS. The absorption coefficients in water were estimated in this case using the method of Gill and von Hippel at 280 nm [34]. We tested this procedure using bovine serum albumin, ovoalbumin, and conalbumin protein standards. The experimental values obtained for dA/dc in our SEC-TD system were 0.67, 0.72, and 1.12 mL·g^−1^·cm^−1^, respectively, which are quite similar to those calculated in silico for these proteins (0.69, 0.73, and 1.11, from the AAS) and reported elsewhere [35]. The values obtained from the calculations for trastuzumab and pertuzumab equal 1.41 and 1.37 mL·g^−1^ at 280 nm, respectively, in close agreement with those measured experimentally.

It is evident that Herceptin and the biosimilars of trastuzumab, namely, Ontruzant and Herzuma, exhibit strikingly similar characteristics across various parameters, including hydrodynamic size, net charge, thermal stability, and extinction coefficient. Notably, the hydrodynamic and electrostatic characterization results indicate a high level of resemblance among the examined mAbs, with minimal variances observed in diffusion coefficients and net charges. Furthermore, the SEC-TD profiles and molecular properties acquired from DLS experiments reveal consistent molecular and physical–chemical characteristics among Herceptin and its biosimilars. The negligible disparities observed in stability and extinction coefficients between Herceptin and the biosimilars underscore their equivalence in terms of structural integrity and biochemical properties. Therefore, based on the evidence presented, it is reasonable to conclude that Herceptin and its biosimilars behave nearly identically, further supporting their interchangeability in clinical practice.

In terms of interactions with HER2, our previous study found that pertuzumab Perjeta has a stronger binding affinity to HER2 than trastuzumab Herceptin [36]. We explained this difference by analyzing the different interfacial contact (IC) descriptors that contribute to the estimated free energy of binding (ΔG_bind_) value using a QSAR model. Additionally, we conducted experiments that revealed that the pertuzumab IgG antibody binds to two HER2 proteins, one per Fab fragment, while trastuzumab mainly forms a monovalent complex. We interpreted this finding using a geometrical model that identified steric crowding in the trastuzumab/HER2 heterotrimer compared to the pertuzumab/HER2 heterotrimer. Now, the result observed in Figure 2 demonstrates that the biosimilars Ontruzant and Herzuma behave exactly the same as Herceptin in their binary interaction, as the concentration and molecular weight profiles are identically replicated, indicating their equivalence in terms of molecular weight and binding affinity to HER2. Small differences in the values for the molecular features (Mw and r_h_) and the previous results obtained in our group can be anticipated [33]. We attribute the small changes to the typical experimental variability in the case of M_w_ and to the slightly different conditions used in the case of r_h_ and [η]. The [η] values are in fact slightly lower now, which is a consequence of the higher temperature used in the current set of experiments (T = 309 K) in comparison to that used in Reference [33]. 

**Figure 6:**
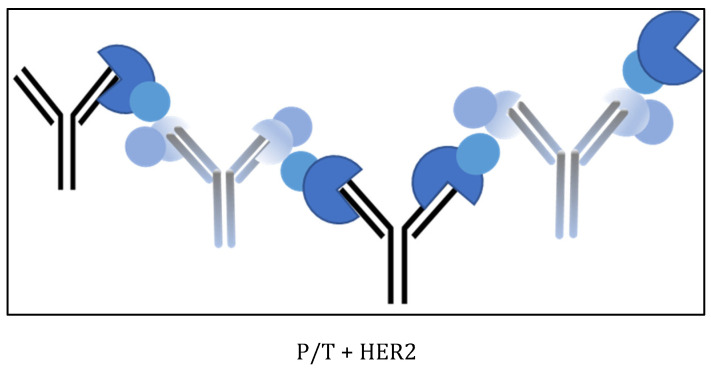
High-order complexes (M_w_~930 kDa) between the mAbs trastuzumab, pertuzumab, and HER2. Pertuzumab again favors C2 complex in trastuzumab/HER2 interaction acting as linkers. HER2 extracellular domains (blue), trastuzumab (T, black), and pertuzumab (P, grey). The different domains of HER2 protein are indicated in different colors as in Figure 3.

Figure 2 and Figure 4 suggest that the biosimilars Ontruzant and Herzuma have a similar capacity as Herceptin for both the binary (C1 and C2) and the ternary (C3) interaction with HER2. The results indicate that they are biologically equivalent to the reference drug to bind HER2 in presence of pertuzumab and in the conditions under study. Notwithstanding, it is interesting to note that when HER2 is added to the previously mixed trastuzumab and pertuzumab antibodies (case 3 in Figure 5), a higher fraction of high-order complexes is formed. In this case, a bivalent trastuzumab should be involved as a linker to form the complex (Figure 6). In that way, the formed complexes of antigens and monoclonal antibodies linked via bivalent binding constitute molecular chains. The formation of such antigen–antibody molecular catenaries was already proposed by Hughes-Jones et al., for the synergistic lysis of red blood cells [37]. The reason for this extremely high avidity of both mAbs together to form these high-order complexes with HER2 is at this moment unknown, but the results suggest an increased availability of trastuzumab Fabs in order to form these complexes, or the appearance of altered intermolecular interactions for binding HER2 to the two mAbs. Further studies are needed to fully understand the mechanisms underlying these observations.

The results obtained in this work settle that the coformulation of mixtures of therapeutic mAbs targeting multiple epitopes represents an appealing strategy to increase their efficacy [38,39] and approach the goal of mimicking the natural polyclonal humoral immune response [40]. Although there are few systematic studies on this approach, reports on mAb mixtures for the treatment of various diseases are emerging [41,42]. At this respect, it is important to understand the potential interactions between mAbs and the effects of variables such as pH, concentration, temperature, and ionic strength [43]. In the specific case of the pertuzumab/trastuzumab mixtures, it should be noted that a fixed-dose combination of pertuzumab and trastuzumab for subcutaneous injection (PHESGO™, F. Hoffmann-La Roche Ltd., Basel, Switzerland) has recently been approved by the U.S. Food and Drug Administration (FDA) and the European Medicines Agency. These findings have important implications for the development of this type therapeutic mAb combinations [44].

While our study provides valuable insights into the similarities between Herceptin and its biosimilars, as well as their interactions with HER2, it is important to recognize certain limitations. Firstly, our investigation focused primarily on physicochemical parameters and binding affinity, leaving out potential functional assays that could provide further understanding of therapeutic efficacy. Additionally, this study was conducted under specific experimental conditions, and variations in factors such as pH, temperature, and ionic strength could influence results. Moving forward, it is beneficial to explore these mAbs’ efficacy in preclinical models or clinical trials, considering real-world scenarios and patient outcomes. Moreover, refining experimental protocols to encompass a broader range of parameters and incorporating functional assays could provide a more comprehensive understanding of the therapeutic potential of these mAbs.

## 4. Materials and Methods

The monoclonal antibodies (mAbs) pertuzumab Perjeta^®^ 450 mg and trastuzumab Herceptin^®^ 150 mg (Roche Pharma AG, Basel, Switzerland), trastuzumab biosimilars Ontruzant^®^ 150 mg (SAMSUNG BIOEPIS, Incheon, South Korea), and Herzuma^®^ 150 mg (Celltrion Healthcare, Jersey City, NJ, USA) were all obtained from University Hospital Ramón y Cajal (Madrid, Spain) through one of the authors (J.C). The human HER2 (10004-HCCH) extracellular domain was purchased from Sino Biological, Inc. Buffer exchange was performed using centrifugal filter units (Amicon^®^ Ultra-0.5 mL (Merck Millipore, Jersey City, NJ, USA). All samples were prepared in buffer 20 mM Tris-Base ULTROL^®^, 150 mM NaCl, pH 7.5. The initial concentrations of HER2 and mAbs were 0.5 and 1.5 mg⋅mL^−1^, respectively.

Dynamic light scattering (DLS) electric field correlations were obtained for the g-eHER2 and mAbs solutions prepared as indicates above, using the Zetasizer Nano ZS (Malvern Instruments, Malvern, UK) at T = 309 K, equipped with disposable cuvettes (Malvern Instruments ZEN0040). By conducting our experiments at this temperature, we aimed to ensure that the experimental conditions closely resemble the in vivo environment where these interactions occur. The electrophoretic mobility (EM) was measured in the Zetasizer Nano ZS apparatus, which uses phase analysis light scattering (PALS). In this application of the technique, a voltage is applied across a pair of electrodes placed at both ends of a disposable capillary cell containing the particle dispersion. Disposable polycarbonate folded capillary cells with gold-plated beryllium–copper electrodes (Malvern Instruments DTS1060) were used to perform the measurements. Charged particles are attracted to the oppositely charged electrode, and their velocity was measured and expressed per unit field strength as the EM, μ_e_. Measurements were carried out in aliquots of the mAbs stock solutions. The measurements were also performed at T = 309 K (36 °C), in samples of c = 5 mg⋅mL^−1^. Finally, we conducted experiments utilizing DLS to investigate the temperature resistance displayed by the mAbs. By subjecting the samples to varying temperatures, between 293 K (20 °C) to 353 K (80 °C), and monitoring changes in their hydrodynamic size using DLS, we aimed to evaluate how variations in temperature may impact the structural integrity and stability of the mAbs studied. This temperature range encompasses conditions relevant to both storage and potential physiological fluctuations, thereby providing valuable insights into the thermal stability profile of the tested formulations. Readers are referred to Supplementary Material for more details about the basic characterization of the mAbs.

The analysis of the binding between mAbs and HER2 receptor and the study of the formed complexes was conducted using SEC-TD. The SEC system used was a GPC-TDAmax (Malvern Instruments) equipped with a Superose™ 6 increase 10/300 GL column (Cytiva, Marlborough, MA, USA). The column was equilibrated at T = 309 K with a buffer consisting of 20 mM Tris Base Ultrol^®^, 150 mM NaCl, pH 7.5. Samples of 100 μL were injected into the SEC column and eluted with the buffer at a flow rate of 0.5 mL·min^−1^. The elution profiles were followed by UV–photodiode array (UV-PDA), differential refractometer (RI), 7° low-angle light scattering detector (LALS), and 90° right-angle light scattering detector (RALS). The data were acquired and analyzed using OmniSEC4.6 software, which determined the absolute molecular weight, M_w_; intrinsic viscosity, [η]; specific absorption coefficient, dA/dc; and concentration, c, of each sample. The extinction coefficient of each species was obtained by matching the concentration measured by means of the RI detector area to that obtained from the UV detector at 280 nm. The analysis was performed maintaining the stoichiometry relation of the reaction at 3:1 in all cases. Three experiments were conducted as follows: (1) HER2 was added over trastuzumab. After 30 min, pertuzumab was added in the same proportion, and the sample was injected after an additional 30 min. (2) HER2 was added over pertuzumab, and after 30 min, trastuzumab was added to the last solution. Conditions and ratio of amounts added were the same as in the first experiment. (3) A solution of trastuzumab and pertuzumab 1:1 was prepared with a concentration of 1 mg·mL^−1^. Over this mixture, HER2 was added. Bovine serum albumin (Sigma Aldrich, St. Louis, MO, USA) was used as a standard reference protein of known molecular weight, concentration, and refractive index increment (dn/dc = 0.185 mL·g^−1^). Before each determination, a BSA solution at a concentration of 2 mg·mL^−1^ was used as a standard, allowing for the determination of molecular weight, concentrations, intrinsic viscosity, and extinction coefficients of the mAbs, HER2 protein, and the complexes.

## 5. Conclusions

In conclusion, the comprehensive characterization and comparative analysis presented in this study shed light on the remarkable similarity between Herceptin and its biosimilars, Ontruzant and Herzuma, across various physicochemical parameters. The findings underscore the interchangeability of these therapeutic agents, offering promising implications for clinical practice. Moreover, our investigation into the interactions with HER2 highlights the equivalence of biosimilars in binding affinity, further supporting their effectiveness in targeted therapy. The observed capacity of biosimilars to form high-order complexes, particularly in combination with pertuzumab, underscores their potential in enhancing therapeutic efficacy. Additionally, our study underscores the potential of coformulated mixtures of therapeutic monoclonal antibodies to increase efficacy, offering a strategy akin to the natural humoral immune response. These insights contribute to the growing body of knowledge surrounding monoclonal antibody therapeutics and pave the way for future developments in the field. 

## Figures and Tables

**Table 1:** Molecular and hydrodynamic properties of the mAbs and target HER2; molecular weight (M_w_), intrinsic viscosity ([η]), hydrodynamic radius (r_h_), and UV coefficient of absorption at 280 nm (dA/dc).

Sample	M_w_ (kDa)	[η] 10^2^ (cm^3^·g^−1^) s.d. ± 0.2	r_h_ (nm) s.d. ± 0.1	dA/dc (g^−1^·mL·cm^−1^) s.d. ± 0.02
Perjeta	147.1 ± 0.8	6.4	5.5	1.33
Herceptin	147.0 ± 1.6	6.5	5.5	1.38
Herzuma	147.9 ± 1.4	6.3	5.5	1.38
Ontruzant	147.7 ± 1.3	6.4	5.5	1.37
g-eHER2	86.3 ± 1.0	6.5	4.4	0.90

Standard deviation values have been obtained from 5 to 10 independent measurements under the same conditions. Theoretical molecular weight values for the mAbs are 145.4 and 145.2 kDa for trastuzumab and pertuzumab, respectively. The experimental value obtained for g-eHER2 includes glycosylation.

**Table 2:** Molecular and hydrodynamic properties of the complexes (case 1); molecular weight, intrinsic viscosity, hydrodynamic radius, and UV coefficient of absorption at 280 nm.

Sample C1/C2	M_w_ (kDa)	[η] 10^2^ (cm^3^·g^−1^) s.d. ± 0.2	r_h_ (nm) s.d. ± 0.1	dA/dc (g^−1^·mL·cm^−1^) s.d. ± 0.02
HRC/HER2	235.7/310.8	6.7/7.9	6.2/7.1	1.22/1.15
ONT/HER2	237.5/313.6	6.9/7.7	6.2/7.2	1.23/1.14
HZM/HER2	238.2/315.7	6.8/7.8	6.2/7.2	1.22/1.15
**C3**		Case 1: TZM/HER2/PZM		
HRC/HER2/PJT	482.5	8.1	8.5	1.22
ONT/HER2/PJT	483.2	8.2	8.6	1.22

Standard deviations are obtained from the values of M_w_ by matching concentration in RI and UV detectors. Theoretical M_w_ is around for C1, C2, and C3 are 234, 320, and 464 kDa, respectively, by assuming the values of M_w_ in Table 1. HRC, ONT, HZM, and PJT stand for Herceptin, Ontruzant, Herzuma, and Perjeta, respectively.

**Table 3:** Molecular and hydrodynamic properties of the complexes (case 2); molecular weight, intrinsic viscosity, hydrodynamic radius, and UV coefficient of absorption at 280 nm.

Sample C2	M_w_ (kDa)	[η] 10^2^ (cm^3^·g^−1^) s.d. ± 0.2	r_h_ (nm) s.d. ± 0.1	dA/dc (g^−1^·mL·cm^−1^) s.d. ± 0.02
PJT/HER2	310.0	8.0	7.4	1.12
**C3**		Case 2: PZM/HER2/TZM		
PJT/HER2/HRC	484.6	8.1	8.5	1.22
PJT/HER2/ONT	481.8	8.2	8.5	1.23
PJT/HER2/HZM	491.6	8.1	8.6	1.22

Standard deviations are obtained from the values of M_w_ by matching concentration in RI and UV detectors. Theoretical M_w_ is around for C1, C2, and C3 are 234, 320, and 464 kDa, respectively, by assuming the values of M_w_ in Table 1.

**Table 4:** Molecular and hydrodynamic properties of the complexes (case 3); molecular weight, intrinsic viscosity, hydrodynamic radius, and UV coefficient of absorption at 280 nm.

**Sample**	**M_w_** **(kDa)**	**[η] 10^2^** **(cm^3^·g^−1^)** **s.d. ± 0.2**	**r_h_** **(nm)** **s.d. ± 0.1**	**dA/dc** **(g^−1^·mL·cm^−1^)** **s.d. ± 0.02**
**C3**		Case 3: TZM/PZM/HER2		
PJT-HRC/HER2	932.0/489.0	9.8/8.0	9.5/8.7	1.23/1.23
PJT-ONT/HER2	945.0/489.0	n.d./8.2	n.d./8.8	1.25/1.25

Standard deviations are obtained from the values of M_w_ by matching concentration in RI and UV detectors of the corresponding peaks. Theoretical M_w_ is around for C1, C2, and C3 are 234, 320, and 464 kDa, respectively, by assuming the values of M_w_ in Table 1.

## Data Availability

The data presented in this study are available upon request from the corresponding author.

## Supplementary Materials

The following supporting information can be downloaded at: https://www.mdpi.com/article/10.3390/ijms25073940/s1, refs. [45,46,47,48,49,50,51,52,53,54,55] cited in Supplementary Materials.

## References

[1] Swain S.M., Shastry M., Hamilton E. (2023). Targeting HER2-Positive Breast Cancer: Advances and Future Directions. Nat. Rev. Drug Discov..

[2] Giordano S.H., Temin S., Davidson N.E. (2018). Systemic Therapy for Patients with Advanced Human Epidermal Growth Factor Receptor 2-Positive Breast Cancer: ASCO Clinical Practice Guideline Update Summary. J. Oncol. Pract..

[3] Hudis C.A. (2007). Trastuzumab—Mechanism of Action and Use in Clinical Practice. N. Engl. J. Med..

[4] De Mattos-Arruda L., Cortes J. (2013). Use of Pertuzumab for the Treatment of HER2-Positive Metastatic Breast Cancer. Adv. Ther..

[5] Von Minckwitz G., Procter M., De Azambuja E., Zardavas D., Benyunes M., Viale G., Suter T., Arahmani A., Rouchet N., Clark E. (2017). Adjuvant Pertuzumab and Trastuzumab in Early Her2-Positive Breast Cancer. N. Engl. J. Med..

[6] Jagosky M., Tan A.R. (2021). Combination of Pertuzumab and Trastuzumab in the Treatment of Her2-Positive Early Breast Cancer: A Review of the Emerging Clinical Data. Breast Cancer Targets Ther..

[7] Liu X., Fang Y., Li Y., Li Y., Qi L., Wang X. (2022). Pertuzumab Combined with Trastuzumab Compared to Trastuzumab in the Treatment of HER2-Positive Breast Cancer: A Systematic Review and Meta-Analysis of Randomized Controlled Trials. Front. Oncol..

[8] Baselga J., Cortés J., Kim S.-B., Im S.-A., Hegg R., Im Y.-H., Roman L., Pedrini J.L., Pienkowski T., Knott A. (2012). Pertuzumab plus Trastuzumab plus Docetaxel for Metastatic Breast Cancer. N. Engl. J. Med..

[9] Swain S.M., Baselga J., Kim S.-B., Ro J., Semiglazov V., Campone M., Ciruelos E., Ferrero J.-M., Schneeweiss A., Heeson S. (2015). Pertuzumab, Trastuzumab, and Docetaxel in HER2-Positive Metastatic Breast Cancer. N. Engl. J. Med..

[10] Krop I.E., Modi S., LoRusso P.M., Pegram M., Guardino E., Althaus B., Lu D., Strasak A., Elias A. (2016). Phase 1b/2a Study of Trastuzumab Emtansine (T-DM1), Paclitaxel, and Pertuzumab in HER2-Positive Metastatic Breast Cancer. Breast Cancer Res..

[11] Miller K.D., Diéras V., Harbeck N., Andre F., Mahtani R.L., Gianni L., Albain K.S., Crivellari D., Fang L., Michelson G. (2014). Phase IIa Trial of Trastuzumab Emtansine with Pertuzumab for Patients with Human Epidermal Growth Factor Receptor 2-Positive, Locally Advanced, or Metastatic Breast Cancer. J. Clin. Oncol..

[12] Urruticoechea A., Rizwanullah M., Im S.-A., Sánchez Ruiz A.C., Láng I., Tomasello G., Douthwaite H., Crnjevic T.B., Heeson S., Eng-Wong J. (2017). Randomized Phase III Trial of Trastuzumab plus Capecitabine with or without Pertuzumab in Patients with Human Epidermal Growth Factor Receptor 2-Positive Metastatic Breast Cancer Who Experienced Disease Progression during or after Trastuzumab-Based Therapy. J. Clin. Oncol..

[13] Rimawi M., Ferrero J.-M., De La Haba-Rodriguez J., Poole C., De Placido S., Osborne C.K., Hegg R., Easton V., Wohlfarth C., Arpino G. (2018). First-Line Trastuzumab plus an Aromatase Inhibitor, with or without Pertuzumab, in Human Epidermal Growth Factor Receptor 2-Positive and Hormone Receptor-Positive Metastatic or Locally Advanced Breast Cancer (PERTAIN): A Randomized, Open-Label Phase II Trial. J. Clin. Oncol..

[14] Perez E.A., Barrios C., Eiermann W., Toi M., Im Y.-H., Conte P., Martin M., Pienkowski T., Pivot X.B., Burris H.A. (2019). Trastuzumab Emtansine with or without Pertuzumab versus Trastuzumab with Taxane for Human Epidermal Growth Factor Receptor 2–Positive Advanced Breast Cancer: Final Results from MARIANNE. Cancer.

[15] Patel T.A., Ensor J.E., Creamer S.L., Boone T., Rodriguez A.A., Niravath P.A., Darcourt J.G., Meisel J.L., Li X., Zhao J. (2019). A Randomized, Controlled Phase II Trial of Neoadjuvant Ado-Trastuzumab Emtansine, Lapatinib, and Nab-Paclitaxel versus Trastuzumab, Pertuzumab, and Paclitaxel in HER2-Positive Breast Cancer (TEAL Study). Breast Cancer Res..

[16] Swain S.M., Miles D., Kim S.-B., Im Y.-H., Im S.-A., Semiglazov V., Ciruelos E., Schneeweiss A., Loi S., Monturus E. (2020). Pertuzumab, Trastuzumab, and Docetaxel for HER2-Positive Metastatic Breast Cancer (CLEOPATRA): End-of-Study Results from a Double-Blind, Randomised, Placebo-Controlled, Phase 3 Study. Lancet Oncol..

[17] Xu B., Li W., Zhang Q., Li Q., Wang X., Li H., Sun T., Yin Y., Zheng H., Feng J. (2023). Pertuzumab, Trastuzumab, and Docetaxel for Chinese Patients with Previously Untreated HER2-Positive Locally Recurrent or Metastatic Breast Cancer (PUFFIN): Final Analysis of a Phase III, Randomized, Double-Blind, Placebo-Controlled Study. Breast Cancer Res. Treat..

[18] Phillips G.D.L., Fields C.T., Li G., Dowbenko D., Schaefer G., Miller K., Andre F., Burris III H.A., Albain K.S., Harbeck N. (2014). Dual Targeting of HER2-Positive Cancer with Trastuzumab Emtansine and Pertuzumab: Critical Role for Neuregulin Blockade in Antitumor Response to Combination Therapy. Clin. Cancer Res..

[19] Wang C., Chen J., Xu X., Hu X., Kong D., Liang G., Wang X. (2020). Dual HER2 Blockade in Neoadjuvant Treatment of HER2+ Breast Cancer: A Meta-Analysis and Review. Technol. Cancer Res. Treat..

[20] Triantafyllidi E., Triantafillidis J.K. (2022). Systematic Review on the Use of Biosimilars of Trastuzumab in HER2+ Breast Cancer. Biomedicines.

[21] Waller C.F., Möbius J., Fuentes-Alburo A. (2021). Intravenous and Subcutaneous Formulations of Trastuzumab, and Trastuzumab Biosimilars: Implications for Clinical Practice. Br. J. Cancer.

[22] Janjigian Y.Y., Bissig M., Curigliano G., Coppola J., Latymer M. (2018). Talking to Patients about Biosimilars. Future Oncol..

[23] Miller E.M., Schwartzberg L.S. (2019). Biosimilars for Breast Cancer: A Review of HER2-Targeted Antibodies in the United States. Ther. Adv. Med. Oncol..

[24] Lamb Y.N. (2018). SB3 (Ontruzant^®^): A Trastuzumab Biosimilar. BioDrugs.

[25] Park J.H., Yeo J.H., Kim Y.S., Park I., Ahn H.K., Cho E.K., Shin D.B., Yang J.Y., Kim H.S., Lee W.K. (2022). Efficacy and Safety of Trastuzumab Biosimilar (CT-P6) Compared With Reference Trastuzumab in Patients With HER2-Positive Advanced Gastric Cancer A Retrospective Analysis. Am. J. Clin. Oncol. Cancer Clin. Trials.

[26] Oda M., Uchiyama S., Noda M., Nishi Y., Koga M., Mayanagi K., Robinson C.V., Fukui K., Kobayashi Y., Morikawa K. (2009). Effects of Antibody Affinity and Antigen Valence on Molecular Forms of Immune Complexes. Mol. Immunol..

[27] Wen J., Arakawa T., Wypych J., Langley K.E., Schwartz M.G., Philo J.S. (1997). Chromatographic Determination of Extinction Coefficients of Non-Glycosylated Proteins Using Refractive Index (RI) and UV Absorbance (UV) Detectors: Applications for Studying Protein Interactions by Size Exclusion Chromatography with Light-Scattering, UV, and RI Detectors. Tech. Protein Chem..

[28] Arakawa T., Wen J. (2001). Size-Exclusion Chromatography with on-Line Light Scattering. Curr. Protoc. Protein Sci..

[29] Mayer C.L., Snyder W.K., Swietlicka M.A., Vanschoiack A.D., Austin C.R., McFarland B.J. (2009). Size-Exclusion Chromatography Can Identify Faster-Associating Protein Complexes and Evaluate Design Strategies. BMC Res. Notes.

[30] Bai Y. (2015). Detecting Protein-Protein Interactions by Gel Filtration Chromatography. Protein-Protein Interactions: Methods and Applications.

[31] Goyon A., Fekete S., Beck A., Veuthey J.-L., Guillarme D. (2018). Unraveling the Mysteries of Modern Size Exclusion Chromatography—The Way to Achieve Confident Characterization of Therapeutic Proteins. J. Chromatogr. B Analyt. Technol. Biomed. Life Sci..

[32] Vega J.F., Ramos J., Cruz V.L., Vicente-Alique E., Sánchez-Sánchez E., Sánchez-Fernández A., Wang Y., Hu P., Cortés J., Martínez-Salazar J. (2017). Molecular and Hydrodynamic Properties of Human Epidermal Growth Factor Receptor HER2 Extracellular Domain and Its Homodimer: Experiments and Multi-Scale Simulations. Biochim. Biophys. Acta Gen. Subj..

[33] Ramos J., Vega J.F., Cruz V., Sanchez-Sanchez E., Cortes J., Martinez-Salazar J. (2019). Hydrodynamic and Electrophoretic Properties of Trastuzumab/HER2 Extracellular Domain Complexes as Revealed by Experimental Techniques and Computational Simulations. Int. J. Mol. Sci..

[34] Gill S.C., von Hippel P.H. (1989). Calculation of Protein Extinction Coefficients from Amino Acid Sequence Data. Anal. Biochem..

[35] Pace C.N., Vajdos F., Fee L., Grimsley G., Gray T. (1995). How to Measure and Predict the Molar Absorption Coefficient of a Protein. Protein Sci..

[36] Cruz V.L., Souza-Egipsy V., Gion M., Pérez-García J., Cortes J., Ramos J., Vega J.F. (2023). Binding Affinity of Trastuzumab and Pertuzumab Monoclonal Antibodies to Extracellular HER2 Domain. Int. J. Mol. Sci..

[37] Hughes-Jones N.C., Gorick B.D., Howard J.C. (1983). The Mechanism of Synergistic Complement-Mediated Lysis of Rat Red Cells by Monoclonal IgG Antibodies. Eur. J. Immunol..

[38] Robak T. (2013). The Emerging Therapeutic Role of Antibody Mixtures. Expert Opin. Biol. Ther..

[39] Raju T.S., Strohl W.R. (2013). Potential Therapeutic Roles for Antibody Mixtures. Expert Opin. Biol. Ther..

[40] Larbouret C., Gros L., Pèlegrin A., Chardès T. (2021). Improving Biologics’ Effectiveness in Clinical Oncology: From the Combination of Two Monoclonal Antibodies to Oligoclonal Antibody Mixtures. Cancers.

[41] Skartved N.J.Ø., Jacobsen H.J., Pedersen M.W., Jensen P.F., Sen J.W., Jørgensen T.K., Hey A., Kragh M. (2011). Preclinical Pharmacokinetics and Safety of Sym004: A Synergistic Antibody Mixture Directed against Epidermal Growth Factor Receptor. Clin. Cancer Res..

[42] Meng Q., Garcia-Rodriguez C., Manzanarez G., Silberg M.A., Conrad F., Bettencourt J., Pan X., Breece T., To R., Li M. (2012). Engineered Domain-Based Assays to Identify Individual Antibodies in Oligoclonal Combinations Targeting the Same Protein. Anal. Biochem..

[43] Singh P., Roche A., Van Der Walle C.F., Uddin S., Du J., Warwicker J., Pluen A., Curtis R. (2019). Determination of Protein-Protein Interactions in a Mixture of Two Monoclonal Antibodies. Mol. Pharm..

[44] Wang B., Deng R., Hennig S., Badovinac Crnjevic T., Kaewphluk M., Kågedal M., Quartino A.L., Girish S., Li C., Kirschbrown W.P. (2021). Population Pharmacokinetic and Exploratory Exposure–Response Analysis of the Fixed-Dose Combination of Pertuzumab and Trastuzumab for Subcutaneous Injection in Patients with HER2-Positive Early Breast Cancer in the FeDeriCa Study. Cancer Chemother. Pharmacol..

[45] Yadav S., Liu J., Shire S.J., Kalonia D.S. (2010). Specific interactions in high concentration antibody solutions resulting in high viscosity. J. Pharm. Sci..

[46] Saito S., Hasegawa J., Kobayashi N., Kishi N., Uchiyama S., Fukui K. (2012). Behavior of Monoclonal Antibodies: Relation between the Second Virial Coefficient (B2) at Low Concentrations and Aggregation Propensity and Viscosity at High Concentrations. Pharm. Res..

[47] Barnett G.V., Qi W., Amin S., Lewis E.N., Razinkov V.I., Kerwin B.A., Liu Y., Roberts C.J. (2015). Structural Changes and Aggregation Mechanisms for AntiStreptavidin IgG1 at Elevated Concentration. J. Phys. Chem. B.

[48] Jaccoulet E., Boccard J., Taverna M., Azevedos A.S., Rudaz S., Smadja C. (2016). High-throughput identification of monoclonal antibodies after compounding by UV spectroscopy coupled to chemometrics analysis. Anal. Bioanal. Chem..

[49] Vermeer A.W.P., Norde W. (2000). The Thermal Stability of Immunoglobulin: Unfolding and Aggregation of a Multi-Domain Protein. Biophys. J..

[50] Le Basle Y., Chenell P., Tokhadze N., Astier A., Sautou V. (2020). Physicochemical Stability of Monoclonal Antibodies: A Review. J. Pharm. Sci..

[51] Lehermayr C., Mahler H.-C., Mäder K., Fischer S. (2011). Assessment of Net Charge and Protein–Protein Interactions of Different Monoclonal Antibodies. J. Pharm. Sci..

[52] Roberts D., Keeling R., Tracka M., van der Walle C.F., Uddin S., Warwicker J., Curtis R. (2014). The Role of Electrostatics in Protein–Protein Interactions of a Monoclonal Antibody. Mol. Pharm..

[53] Kiraga J., Mackiewicz P., Mackiewicz D., Kowalczuk M., Biecek P., Polak N., Smolarczyk K., Dudek M.R., Cebrat S. (2007). The relationships between the isoelectric point and: Length of proteins, taxonomy and ecology of organisms. BMC Genom..

[54] Knight C.G., Kassen R., Hebestreit H., Rainey P.B. (2004). Global analysis of predicted proteomes: Functional adaptation of physical properties. Proc. Natl. Acad. Sci. USA.

[55] Wang M., Zhu D., Zhu J., Nussinov R., Ma B. (2018). Local and Global Anatomy of Antibody-Protein Antigen Recognition. J. Mol. Recognit..

