# Supplementary material for: Exploring the Combined Action of Adding Pertuzumab to Branded Trastuzumab versus Trastuzumab Biosimilars for Treating HER2+ Breast Cancer
Source: Int J Mol Sci. 2024 Apr 1;25(7):3940. doi: 10.3390/ijms25073940 (PMC11011846; doi:10.3390/ijms25073940)
Supplement: Supplementary file 1 [file ijms-25-03940-s001.zip › ijms-2907519-supplementary.pdf]

Exploring the Combined Action of Adding Pertuzumab to Branded Trastuzumab versus Trastuzumab Biosimilars for Treating HER2+ Breast Cancer

Emma Franco-Mateos [1], Virginia Souza-Egipsy [1], Laura García-Estévez [2], José Pérez-García [3,4,5], María Gion [6], Laia Garrigós [3], Patricia Cortez [7], Cristina Saavedra [6], Patricia Gómez [3], Carolina Ortiz [3], Víctor L. Cruz [1], Javier Ramos [1], Javier Cortés [3,4,8,†] and Juan F. Vega [1,*]

[1] BIOPHYM, Department of Macromolecular Physics, Instituto de Estructura de la Materia, IEM-CSIC, C/Serrano 113 bis, 28006 Madrid, Spain; emmafranco00@gamil.com (E.F.-M.); virginia.souza-egipsy@csic.es (V.S.-E.); vl.cruz@csic.es (V.L.C.); j.ramos@csic.es (J.R.)
[2] Breast Cancer Department, MD Anderson Cancer Center, 28033 Madrid, Spain; lgestevez@mdanderson.es
[3] International Breast Cancer Center (IBCC), Pangaea Oncology, Quiron Hospital, 08017 Barcelona, Spain; josemanuel.perez@ibcc.clinic (J.P.-G.); laia.garrigos@ibcc.clinic (L.G.); patricia.gomez@ibcc.clinic (P.G.); carolina.ortiz@ibcc.clinic (C.O.); javier.cortes@maj3.health (J.C.)
[4] Medica Scientia Innovation Research (MedSIR), 08018 Barcelona, Spain
[5] Medica Scientia Innovation Research (MedSIR), Ridgewood, NJ 07450, USA
[6] Medical Oncology Department, Ramón y Cajal University Hospital, 28034 Madrid, Spain; mariagion@gmail.com (M.G.); cristina.saavedra@iobmadrid.com (C.S.)
[7] IOB, Institute of Oncology, 28007 Madrid, Spain; patricia.cortez@iobmadrid.com
[8] Faculty of Biomedical and Health Sciences, Department of Medicine, Universidad Europea de Madrid, 28670 Madrid, Spain
* Correspondence: jf.vega@csic.es
† J.C. was at Vall d'Hebron Institute of Oncology, 08035 Barcelona, Spain, during the development of this work.

Supplementary Material

Dynamic Light Scattering (DLS) technique allowed us to extract the diffusion coefficient, D_s, from the autocorrelation function. Hydrodynamic size, r_h, has been obtained from the results obtained for D_s, the diffusion coefficient at infinite dilution, using the Stokes-Einstein equation:

$$D_S = \frac{k_B T}{6\pi\eta r_h} \quad (1)$$

with k_B, the Boltzmann constant and η the buffer viscosity at T = 309 K. The results obtained for r_h are shown in Tables 1-4 of the main manuscript.

DLS results are presented in Figure S1 as the squared electric field autocorrelation function, $[g_1(t)]^2$, for the samples at T = 309 K (36 °C) and a selected concentration of c = 2 mg·mL^{-1}. Although it is well-known that mAbs are prone to aggregation, all samples are free of aggregates in the conditions used in this study. Results have been obtained in the concentration range from 1 to 10 mg·mL^{-1}. From

the results, the mutual diffusion coefficient (D_m) has been obtained by cumulant analysis and represented versus concentration (Figure S2).

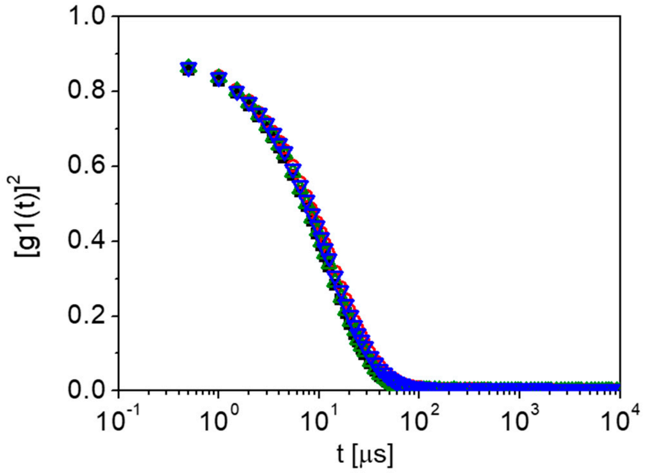

Figure S1. Averaged squared electric field time autocorrelation function, $[g_1(t)]^2$, of the mAbs studied at a concentration of 2 mg·mL^{-1}. Perjeta (black), Herceptin (red), Ontruzant (blue) and Herzuma (green).

The k_D values determined from the slope of the representation provide an assessment of protein-protein interactions. In particular, a positive k_D is a result of an increase in D_m over D_s with increasing concentration, whereas a negative k_D is a result of decreasing D_m as the solution concentration increases. Thus, a positive k_D signifies repulsive interactions, and a negative k_D implies attractive intermolecular interactions. In the conditions studied, there are attractive interactions among the molecules, in agreement with reported values in the literature for different mAbs in similar conditions.[45-48] it should be noted that within the experimental error, the value of k_D is nearly the same in all the cases. In the case of pertuzumab sample a slightly lower value of k_D is observed, which likely comes from the different AAS in this case, giving rise to slightly different degree of molecular interactions.

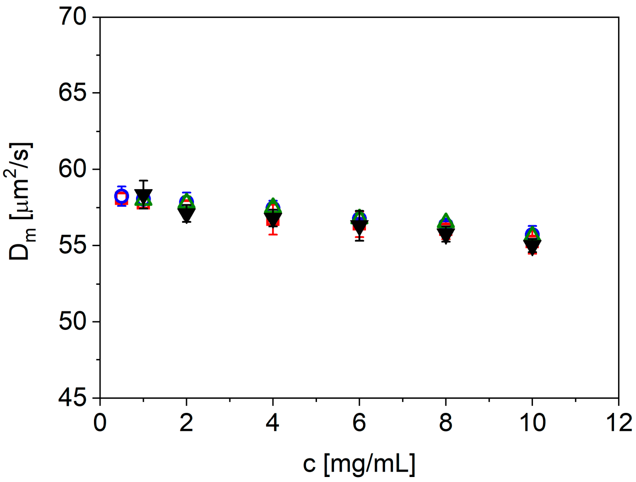

Fig. S2. Mutual diffusion coefficient (D_m) for the mAbs at pH 7.0 and 150 mM NaCl solution ionic strength. The slope and intercept represent $D_s k_D$ and D_s (self-diffusion coefficient), respectively. Code color as in Fig. S1.

The self-diffusion coefficient (D_s) can be obtained at infinite dilution. The r_h values can be obtained by taking the D_s values and the Stokes-Einstein relationship Equation (1). The values of D_s and r_h obtained from the analysis of DLS results are also listed in Table S1. The values obtained for the hydrodynamic size are virtually identical in all the cases, around 5.5 nm, also in agreement with previous results obtained in mAbs.[45-48]

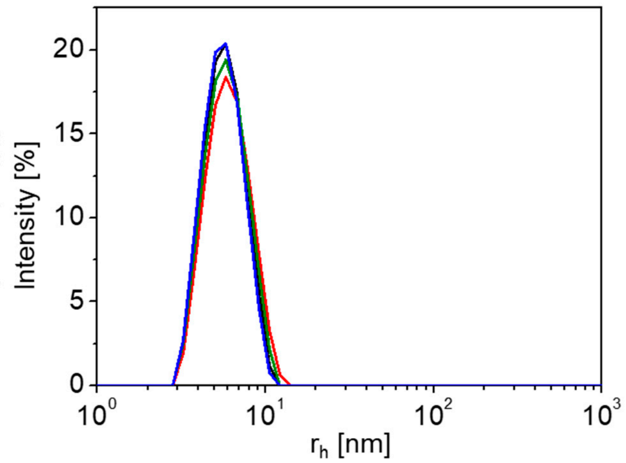

Fig. S3. Size distribution profile of the mAbs studied. Code color as in Fig.S1.

In Figure S3 we can observe the size distribution profile obtained from the autocorrelation function in Figure S2. A narrow distribution and an absence of aggregates characterize the profiles obtained in all the cases. The results in Figures S1 to S3 point towards a high degree of similarity in the samples studied. Both the molecular weight and the diffusion coefficient (hydrodynamic size) differ no more than 2% among the samples, suggesting a similar, if not identical, morphology/shape of the mAbs.

The size distribution profiles obtained by DLS during temperature sweeps from 293 K (20 °C) to 353 K (80 °C) are observed in Figure S4. An increase in the size of the mAbs is seen as temperature increases, which indicates the onset of a denaturation process, giving rise to a loss of higher-order structure through unfolding. The kinetics is nearly identical in all the samples studied. The extracted size distribution from the autocorrelation function indicates a monomodal population that increases in size and polydispersity, as expected from a denaturation process of a protein.

Table S1. Size distribution profile of the samples studied. Code color as in Figure S1.

**Sample**	**$D_s \times 10^7$ [$\mu m^2 \cdot s^{-1}$]**	**k_D [$mL \cdot g^{-1}$]**	**r_h [nm]**
Perjeta	58.2 ± 0.3	-5.5 ± 0.07	5.50 ± 0.03
Herceptin	58.1 ± 0.1	-4.7 ± 0.03	5.51 ± 0.01
Herzuma	58.3 ± 0.1	-4.5 ± 0.04	5.49 ± 0.01
Ontruzant	58.4 ± 0.1	-4.6 ± 0.03	5.48 ± 0.01

Results obtained in 5 to 10 samples. Averaged values and standard deviations indicated

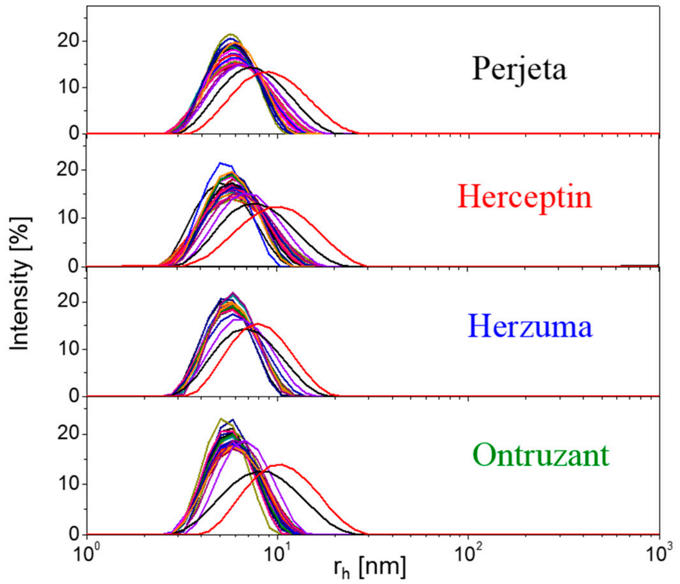

Fig. S4. Size distribution profiles of the mAbs as temperature increases from 293 K to 353 K.

The values of the hydrodynamic radius at each temperature have been determined by means of the application of the cumulant analysis. In Figure S5 the evolution of r_h in the temperature range from 293 K (20 °C) to 353 K (80 °C) is observed. A clear increase of the mAbs hydrodynamic seen is clearly seen above a characteristic temperature of around 338 K (65 °C). Each protein has a specific T_m, which is the temperature where 50% of proteins are unfolded. mAbs have a good stability and resistance to moderate thermal stress, when compared to other proteins.[49,50] However, as multi-domain proteins, the thermal denaturation of antibodies is complex, showing in DSC two endothermic peaks, usually around 338 K (65 °C) and 353 K (80 °C). The denatured state is reached after several, at least partly independent, intermediate states. The results shown in Figure S5 show the characteristic first stages of the denaturation of the mAbs, starting around 338 K (65 °C). No differences have been found among the samples under study.

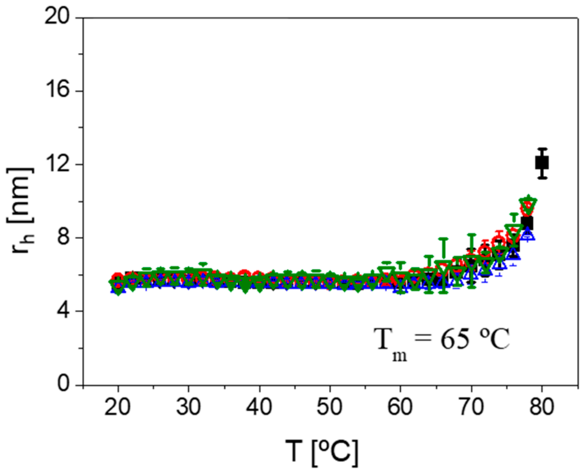

Fig. S5. Hydrodynamic radius versus temperature for the samples under study. Code color as in previous figures. The symbols are the averages if 3 independent measurements.

Table S2. Electrostatic properties of the mAbs under study

**Sample**	**μ_e [μm·cm·$V^{-1}s^{-1}$]**	**ζ [mV]**	**Z**	**Z [ProtCal][a]**
Perjeta	0.25 ± 0.01	2.7 ± 0.2	+5.2 ± 0.4	+6.5
Herceptin	0.40 ± 0.04	4.3 ± 0.3	+8.3 ± 0.8	
Herzuma	0.41 ± 0.02	4.4 ± 0.2	+8.5 ± 0.4	+10.8
Ontruzant	0.40 ± 0.05	4.3 ± 0.4	+8.3 ± 0.9	

[a]Estimated from the AAS using Protein Calculator platform (http://protcalc.sourceforge.net/cgi-bin/protcalc). Measurements performed in 3 independent samples. Averaged and standard deviations indicated.

To study the electeostatic properties of branded mAbs and biosimilars, phase analysis light scattering (PALS) is an especially interesting technique to be applied, as it permits to accurately measure samples with low particle mobility. Due to the low stability of the solutions, the monomodal analysis has been applied. This type of analysis does not produce EM distributions but results in a faster experiment, avoiding sample and electrode degradation. Additionally, reduced voltages have been used (in the range 3 – 5 V). In some case the general-purpose analysis mode has also been used in order to test the quality and reproducibility of the experiments. The measured EM can be used to determine the zeta-potential, ζ, using Henry's equation:

$$\zeta = \frac{3\eta\mu_e}{2\varepsilon f(\kappa a)} \qquad (2)$$

where ε is the dielectric constant of the medium, η is the viscosity of the dispersant, and f(κa) is the Henry's function. If the value ζ of a particle is less than k_BT/e (i.e., 25.7 mV at 298 K), the effective molecular charge can be evaluated using the Debye-Hückel-Henry (DHH) approximation to correct for ionic radii and ionic strength effects:

$$Z = \frac{6\pi\eta a\mu_e(1+\kappa a)}{f(\kappa a)\, e} \qquad (3)$$

where e is the electronic charge, a is the particle radius, and κ the inverse Debye length. This later can be calculated for monovalent salt at any ionic strength, I, and temperature as:

$$\kappa^{-1} = \left(\frac{\varepsilon_0\varepsilon k_B T}{2000e^2 I N_A}\right)^{1/2} \qquad (4)$$

In general, it is considered that the values obtained for the effective charge, Z, will be close to the "true net charge" of the proteins and complexes. However, it should be emphasized that within the framework given by Equations (2) – (4), Z is the fixed charge arising not only from the ionized groups on the protein, but also from the ions bound in the Stern layer.

The estimated values ζ-potential and Z using Equations (3) – (5) in experimental section are listed in Table S2 for the systems under study. For the calculations of Z, the radius of the particle (a) has been taken as the hydrodynamic radius (r_h) obtained from DLS measurements in Table S1. Concerning electrostatic properties in **Table S2**, the reported positive value of Z for the mAbs at pH 7.5 is in the same range as those values reported in other studies for IgG1 antibody at pH within 5.0 and 9.0 and low ionic strength.[51,52] The positive net-charge is typical in antibodies. It has been found that antibodies prefer positive net-charge presumably to help antibody proteins approach negatively charged protein antigens, which are common in proteomes.[53-55] It should be noted the good agreement obtained between experimental results and those calculated from the AAS using the Protein Calculator platform. A clear difference exists between pertuzumab and trastuzumab, as the former shows lower values of the positive net charge, suggestion a lower value of pI. Moreover, no differences have been found among the trastuzumab biosimilars, as it can be seen in Table S2.

References

1. Yadav, S.; Liu, J.; Shire, S.J.; Kalonia, D.S. Specific interactions in high concentration antibody solutions resulting in high viscosity. *J. Pharm. Sci.* **2010**, *99*, 1152–1168. https://doi.org/10.1002/jps.21898.
2. Saito, S.; Hasegawa, J.; Kobayashi, N.; Kishi, N.; Uchiyama, S.; Fukui, K. Behavior of Monoclonal Antibodies: Relation between the Second Virial Coefficient (B2) at Low Concentrations and Aggregation Propensity and Viscosity at High Concentrations. *Pharm. Res.* **2012**, *29*, 397–410. https://doi.org/10.1007/s11095-011-0563-x.
3. Barnett, G.V.; Qi, W.; Amin, S.; Lewis, E.N.; Razinkov, V.I.; Kerwin, B.A.; Liu, Y.; Roberts, C.J. Structural Changes and Aggregation Mechanisms for AntiStreptavidin IgG1 at Elevated Concentration. *J. Phys. Chem. B* **2015**, *119*, 15150−15163. https://doi.org/10.1021/acs.jpcb.5b08748.
4. Jaccoulet, E.; Boccard, J.; Taverna, M.; Azevedos, A.S.; Rudaz, S.; Smadja, C. High-throughput identification of monoclonal antibodies after compounding by UV spectroscopy coupled to chemometrics analysis. *Anal. Bioanal. Chem.* **2016**, *408*, 5915–5924. https://doi.org/10.1007/s00216-016-9708-4.
5. Vermeer, A.W.P.; Norde, W. The Thermal Stability of Immunoglobulin: Unfolding and Aggregation of a Multi-Domain Protein. *Biophys. J.* **2000**, *78*, 394–404. https://doi.org/10.1016%2FS0006-3495(00)76602-1.
6. Le Basle, Y.; Chenell, P.; Tokhadze, N.; Astier, A.; Sautou, V. Physicochemical Stability of Monoclonal Antibodies: A Review. *J. Pharm. Sci.* **2020**, *109*, 169–190. https://doi.org/10.1016/j.xphs.2019.08.009.
7. Lehermayr, C.; Mahler, H.-C.; Mäder, K.; Fischer, S. Assessment of Net Charge and Protein–Protein Interactions of Different Monoclonal Antibodies. *J. Pharm. Sci.* **2011**, *100*, 2551–2562. https://doi.org/10.1002/jps.22506.
8. Roberts, D.; Keeling, R.; Tracka, M.; van der Walle, C.F.; Uddin, S.; Warwicker, J.; Curtis, R. The Role of Electrostatics in Protein–Protein Interactions of a Monoclonal Antibody. *Mol. Pharm.* **2014**, *11*, 2475–2489. https://doi.org/10.1021/mp5002334.
9. Kiraga, J.; Mackiewicz, P.; Mackiewicz, D.; Kowalczuk, M.; Biecek, P.; Polak, N.; Smolarczyk, K.; Dudek, M.R.; Cebrat, S. The relationships between the isoelectric point and: Length of proteins, taxonomy and ecology of organisms. *BMC Genom.* **2007**, *8*, 163. https://doi.org/10.1186/1471-2164-8-163.
10. Knight, C.G.; Kassen, R.; Hebestreit, H.; Rainey, P.B. Global analysis of predicted proteomes: Functional adaptation of physical properties. *Proc. Natl. Acad. Sci. USA* **2004**, *101*, 8390–8395. https://doi.org/10.1073/pnas.0307270101.
11. Wang, M.; Zhu, D.; Zhu, J.; Nussinov, R.; Ma, B. Local and Global Anatomy of Antibody-Protein Antigen Recognition. *J. Mol. Recognit.* **2018**, *31*, e2693. https://doi.org/10.1002/jmr.2693.
